# Commencement of Medical Lectures in the University of Buffalo

**Published:** 1863-11

**Authors:** 


### COMMENCEMENT OF MEDICAL LECTURES IN THE UNIVERSITY OF BUFFALO.

The first Lecture of the season at the *Buffalo Medical College*, was given on Wednesday, Nov. 5th, by Professor James P. White, who chose for a topic, as introductory to his course, The History of Midwifery. Commencing with the derivation of the term, he traced the early history of the art, beginning with Adam and Eve, and making a quotation from a writer of the eighteenth century to show that Adam, yielding to the necessities of the case, must have rendered obstetrical assistance to Eve when she gave birth to Cain, thus giving the department of obstetrics precedence over all others in point of time." This, however, was not regarded as the true time to date the origin of the art, but that period when the rude, but well-meant efforts of one friend to relieve another in such cases closed, or gave place to those who were supposed to have more information and experience, and, consequently, to possess greater skill. The earliest history we possess—the Scriptures—make frequent mention of female practitioners among the Hebrews and Egyptians; yet, it was not, perhaps, possible to make accurate date of the commencement of the practice by the Egyptian and Hebrew Midwives.

It was remarked that in all ages, and in every country (China, perhaps, excepted) the practice had been in the hands of females down to almost the present era; and both in the ancient and modern language the appellation of the practitioner was always in the feminine, with the exception of the French term Accoucheur.

The early practice of the art was shown to have been exceedingly rude and simple, but to have been practiced in some shape among all nations—barbarous and civilized—though ages may have transpired before it was reduced to the systematic regularity of a science. It would seem probable that this department of medicine was the first, that of necessity, was exercised as an art; and it is quite certain that it is the last to receive the highest state of improvement.

"Hippocrates, who, as you are doubtless aware, practiced medicine 460 years before the Christian era, is styled by some authors the Father of Midwifery, as well as of Physic. It is unnecessary to dwell upon the unbounded veneration with which his name has been regarded by succeeding ages. His works must ever command the highest admiration as carrying with them indubitable proofs of profound and laborious research. His sagacity and experience fully entitle him to the praise of subsequent obstetricians. He must have been possessed of all that was known on the subject, at the time in which he wrote."

A brief account was presented of the imperfect and rude notions entertained by Hippocrates, and also of the difficulties he encountered in obtaining any reliable information since it was not taken from bedside observation. "The precise character of the symptoms to be remedied, and the *ratio medendi* of the means used, came to him through the medium of uneducated females, who were utterly incapable of forming a correct diagnosis or making a just report.

It is not wonderful then that his notions on this subject should now excite a smile, or that we hold in light estimation his uncouth and curious remedies. From the time of Hippocrates until after the commencement of the Christian era, Midwifery made no progress. Celsus, who flourished in the reign of the Emperor Tiberias, A. D., '37, although an ardent admirer and close copyist of Hippocrates, threw some new light on parturition. He was the first to recommend the introduction of the hand to rectify position; and to deliver by the feet where the arm presented, and must, of course, have been aware of the error of Hippocrates in attaching such fatal consequences to foot presentations. Galen was the next name in the catalogue, who appeared 600 years subsequent to the writings of Hippocrates and 150 years after the Christian era. In him we find a most elaborate and able expounder of the works of this great man, and he claims the singular merit of bringing back physicians to the Father of Medicine, from whose simple, but sound principles, he insisted they had been too much divided by the Methodists and other theorists. Although the writings of Galen were chiefly paraphrastic, yet, he certainly effected many improvements; and in the anatomy of the viscera of the female pelvis, he may lay claim to the title of discoverer."

We should be most happy to have been able to follow the lecture to its close, thus offering our readers a condensed history of the rise and progress of obstetrical science down to the present time. We have been able to offer only a mere glance at the first portions and leave the more important teachings for those who were favored with the opportunity of listening. The interest which would naturally attach to a condensed history of Midwifery was greatly increased by the spirit and force of the speaker, who lends his own enthusiasm, in a great degree, to the themes upon which he speaks. On this occasion he was greeted by the appearance in his lecture room, of an unusually large and intelligent class of students, besides many professional friends who were present, to show their interest not only in the speaker and subject, but also in the College whose prosperity and advancement is an object of congratulation and pride.

